# Opening the Black Box of Electronic Health: Collecting, Analyzing, and Interpreting Log Data

**DOI:** 10.2196/resprot.6452

**Published:** 2017-08-07

**Authors:** Floor Sieverink, Saskia Kelders, Mannes Poel, Lisette van Gemert-Pijnen

**Affiliations:** ^1^ Centre for eHealth and Wellbeing Research Department of Psychology, Health and Technology University of Twente Enschede Netherlands; ^2^ Optentia Research Focus Area North-West University Vanderbijlpark South Africa; ^3^ Human Media Interaction group Department of Computer Science University of Twente Enschede Netherlands

**Keywords:** eHealth, black box, evaluation, log data analysis

## Abstract

In electronic health (eHealth) research, limited insight has been obtained on process outcomes or how the use of technology has contributed to the users’ ability to have a healthier life, improved well-being, or activate new attitudes in their daily tasks. As a result, eHealth is often perceived as a black box. To open this black box of eHealth, methodologies must extend beyond the classic effect evaluations. The analyses of log data (anonymous records of real-time actions performed by each user) can provide continuous and objective insights into the actual usage of the technology. However, the possibilities of log data in eHealth research have not been exploited to their fullest extent. The aim of this paper is to describe how log data can be used to improve the evaluation and understand the use of eHealth technology with a broader approach than only descriptive statistics. This paper serves as a starting point for using log data analysis in eHealth research. Here, we describe what log data is and provide an overview of research questions to evaluate the system, the context, the users of a technology, as well as the underpinning theoretical constructs. We also explain the requirements for log data, the starting points for the data preparation, and methods for data collection. Finally, we describe methods for data analysis and draw a conclusion regarding the importance of the results for both scientific and practical applications. The analysis of log data can be of great value for opening the black box of eHealth. A deliberate log data analysis can give new insights into how the usage of the technology contributes to found effects and can thereby help to improve the persuasiveness and effectiveness of eHealth technology and the underpinning behavioral models.

## Introduction

Many electronic health (eHealth) technologies, such as behavior change technologies, aim to support users in reaching certain health-related behavioral outcomes. While such technologies can be effective [1,2], one of the main problems is that users’ adoption and long-term use remains lower than expected [3-5]. Moreover, eHealth research is dominated by a classic conception of medical research where randomized controlled trials (RCTs) are the golden standard for measuring outcomes [6]. Although RCTs provide valuable insight into the effectiveness of an intervention, fundamental to this methodology is to have the technology as a fixed entity for all participants throughout the entire intervention period. In contrast, (eHealth) technology can be characterized by its constant evolution and, consequently, apps or interventions often become obsolete by the time the results of the RCT are available.

Furthermore, to conform to the complexity of behavior change, eHealth technologies often consist of multiple components that may interact in reaching a certain effect and that people can use in many different ways in terms of the elements they use as well as the frequency, time, and place of use [7,8]. However, RCTs only provide insight into outcomes at fixed time points and treat technologies as a singular entity. Therefore, no insight can be obtained on process outcomes or how the use of the different components of the technology has contributed to healthier living, improved well-being, or a user’s ability to conduct daily tasks [7,9,10]. This particular lack of insight is known as the “Black Box Phenomenon” [2,10,11]. To open the black box of eHealth and to investigate why, how, and for whom a certain technology is of the most value, methodologies must extend beyond the classic evaluations of effect only. In other words, the characteristics of eHealth technology change the way evaluations are conducted. In this view, Hekler and colleagues pled for an “agile science” approach that enables early and frequent insight into the process of behavior change via technology [7].

**Figure 1:**
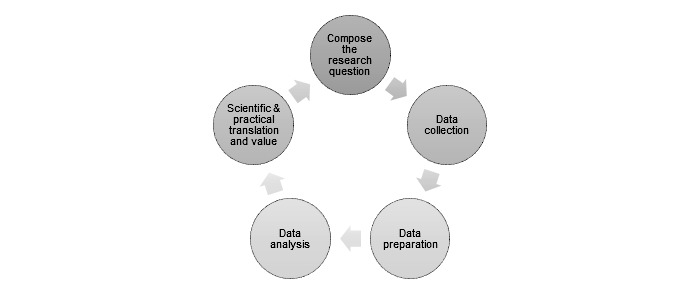
Steps in log data analysis.

The CeHRes Roadmap (Figure 1) adopts an agile approach in the development and evaluation process of eHealth technology. This roadmap is based on an extensive literature review of eHealth frameworks and follows a holistic and participatory research and development approach. The following phases can be distinguished in the development and evaluation of eHealth: (1) contextual inquiry, (2) value specification, (3) design, (4) operationalization, and (5) summative evaluation. The results of each phase should be the subject of formative evaluation in order to collect input for improving the product [4].

According to the roadmap, technology development and evaluation is an iterative, flexible, and dynamic process without a fixed endpoint. In this approach, continuous (formative and summative) evaluation is needed that is interwoven with all stages of technology development. The outcomes of such evaluations will be used for analyzing the process, recognizing the areas of improvement, and diving deeper into the usage (the dose) that is needed to reach certain effects (the response). Thus, technology already in early stages of development will be reshaped by its usage. In order to do so, more advanced methods are needed to understand what people do with eHealth technology and how this is related to the impact.

The analysis of log data, defined as anonymous records of real-time actions performed by each user, has the potential to provide continuous and objective insight into the actual usage (of the different components) of the technology. Such analyses are a promising approach to explain the outcomes of the more traditional methods, such as RCTs, by gaining insight into the mediating mechanisms that contribute to the found effects [8,9] and have also the potential to identify unexpected effects of a technology. Thus, the use of log data fits the aim of eHealth evaluation according to the CeHRes Roadmap by enabling early improvements of the technology in order to improve the evaluation outcomes.

Log data analyses are currently used in diversified domains, such as education [12,13], human-computer interaction [14,15], and network security [16] where they are mainly used to analyze system performance and acceptance based on models for information retrieval. However, technology has evolved and the aim of behavior change technologies is not only to provide information, but rather to stimulate and support people in their process of behavior change [17]. Therefore, information is needed regarding how the use of technology can explain the engagement and involvement of the individual user, the found effects, and how the technology fits the user and the context.

Log data analyses in eHealth research have mainly focused on descriptive statistics, such as the number of logins, time spent, and the frequency of use of the different elements by all users as a group [18-20]. Although these statistics do provide valuable information regarding the usage of the technology, they also assume a dose-response relationship without taking the goal of the user into account. Furthermore, such analyses do not always provide insight into the actual process of technology use in relation to behavior change. For example, a longer and more frequent exposure to an eHealth technology might indicate how well the system fits the users’ needs, but it can also signify unfocused and/or non-strategic use or an inefficient system [9,21]. The same applies to only counting the number of logins, since a user can log out directly without using the technology. As such, measures are needed that indicate how the actual use of the (different elements of the) technology can explain the found effects.

There is evidence that the use of log data can be of added value to the more traditional approaches of eHealth evaluation, but its possibilities have not yet been exploited to their fullest extent or have mostly been described on a conceptual level [7-9]. The aim of this paper is to give a more practical description of how log data can be of added value to understand the use of an eHealth technology with a broader approach than only descriptive statistics. Here, we describe what research questions can be answered using log data. Furthermore, we provide direction on which steps need to be taken in the collection, preparation, and analysis of log data, and how to interpret and apply the results (Figure 1).

The results and ideas presented in this paper are substantiated with examples from prior log data research. They do not provide an exhaustive overview of all research conducted in this field but are used to illustrate the possibilities of log data as a starting point for further research to open the black box of eHealth.

## Methods

### What is Log Data?

Generally (transactional) log data can be seen as behavioral logs that contain information regarding the interactions between a system and the users of that system [9,14,15]. These interactions can include information regarding time of the action, content that is viewed or used, mouse clicks, browsing patterns, or saving information in the system. An important benefit of log data is that it represents the actual and continuous usage behavior and not subjective, recalled behavior.

The use of log data mainly focuses on the interaction with Web-based technologies. However, eHealth technology has evolved from Web- or telephone-based systems that required users’ active involvement into unobtrusive and pervasive systems that are embedded into users’ daily lives. For example, many people currently use lifestyle apps, such as “Runkeeper” or food diaries, to support their daily routines. Wearable devices like the Fitbit, Jawbone, or Apple Watch can continuously collect real-time health-related data for personalized coaching via apps for mobile phones or tablets. To be able to understand as much as possible of what people do, log data should not be limited to technologies and/or how they experience it, but to the actual usage of Web-based technologies including interactions with mobile phone apps and wearables as well.

### Composing Research Questions

Before the log data can be collected and analyzed, an important step is to revisit the goals of the technology and the subsequent research questions. A variety of research questions can be addressed with log data analysis, depending on the type and the goal of the eHealth technology and on the phase of development. According to the CeHRes Roadmap, log data analyses can be of added value in both the formative and summative evaluation phases [4].

Formative evaluation is conducted within and between the different phases of the roadmap. The aim of this type of evaluation is to check whether the goals of that phase have been reached. In the early operationalization phase, for example, log data has the potential to evaluate the use of the system and to assess what people do (or not do) with a technology. Critical moments for dropping out can be identified, as well as profiles for both users and usage. The results from these evaluations can be used to improve an early release of the technology before it will be available for a bigger group of users, which fits the “agile science” approach [7]. Possible research questions for formative system evaluations are shown in Textbox 1.

**Questions for formative system evaluations.**

Questions

- What usage patterns emerge when users navigate through the technology?
- Which (combinations of) elements of the technology are used?
- When do users drop out?
- How do users respond to behavior change strategies and persuasive triggers (strategies to support users in performing certain [usage] behaviors and/or long-term use, such as reminders)?

For example, in a previous study we found that users of a personal health record (PHR), an electronic application consisting of different tools for monitoring and coaching patients with chronic conditions to support self-management [22], followed the global menu structure when exploring the PHR for the first time. Furthermore, most users were likely to drop out when they visited the education section as a first step after the first login [23].

The uptake and impact of a technology are measured during the summative evaluation phase. Impact refers to whether the intended goals of the technology have been realized in terms of behavioral, clinical, and organizational outcomes. Uptake refers to the implementation and usage of the technology. Log data can be used to assess the uptake of the technology. Where log data analyses in the operationalization phase mainly focus on the system performance, in this phase research questions to evaluate the whole of the system, the user, and the context can be formulated. Possible research questions to evaluate the system can be seen in Textbox 2.

**Questions to evaluate the system.**

Questions

- How do users use the technology in order to complete an intervention or to achieve their health-related goals (in terms of frequency or combinations of elements they use)?
- How well do the users adhere to the intended usage of the technology?
  - What are predictors for adherence or dropping-out?
  - How does the use of the technology change over time?
- How did these usage patterns contribute to the (clinical, behavioral and organizational) impact? In other words, what is the dose-response relationship?

For example, studies by Kelders et al [21] and Van Gemert et al [24] showed when users of a Web-based intervention for the early treatment of depressive symptoms (“Living to the Full”) were at risk of dropping out and might need additional support. In another example, Freyne et al [25] found that uploading a profile picture on a diet support site in the first week resulted in higher return rates.

Research questions to evaluate the users of the technology and possible context-related questions are shown in Textbox 3.

**User evaluation and context-related questions.**

Questions

- User evaluation
  Who is motivated and capable of using the eHealth technology?
  - Who are the long-term users?
  - Who are the drop-outs?
- Context-related evaluation
  - How does the responsiveness of caregivers (eg, time until replying to a users’ message) influence the use of eHealth technology by patients?
  - How does the technology integrate into users’ daily lives?

Log data analyses can also provide answers to more fundamental research questions related to existing models and theories [8]. Behavior change theories and behavior models often form the basis for the content and the structure of eHealth technologies. For example, mental health interventions are frequently based on the principles of cognitive behavioral therapy and principles from the goal setting theory are used to support users in reaching their health-related goals. Log data can be used to check whether the incorporated (combination of) elements that represent certain theoretical concepts (eg, a chat box to facilitate social support) have been used. Or, when one of the goals of a technology is to improve self-efficacy, for example, log data can provide insight into what the most effective usage patterns are and for whom to experience any improvements in the self-efficacy. Research questions related to the evaluation of existing models and theories are shown in Textbox 4.

**Research questions to evaluate existing models and theories.**

Questions

- To what extent did the users find and use the (combination of) elements of the technology that represent certain theoretical concepts?
- How did the use of (a combination of) these elements contributed to any improvements in the outcomes?

For example, the “Living to the Full” intervention is based on the principles of acceptance and commitment therapy (ACT). An effect evaluation indicated that a Web-based technology based on ACT might help users reduce depressive complaints [26]. However, a log data analysis revealed that many users of that intervention did not open the mindfulness exercises that are assumed to be an important element of ACT [24]. This might be an indicator that the concept of mindfulness is insufficiently operationalized in the intervention and that the found effects are an underestimation of the attainable effects.

While not complete or exhaustive, this overview serves as a starting point for composing suitable research questions for a holistic and agile evaluation of eHealth technology. The proposed questions can be adjusted based on the goal of the technology and the incorporated behavioral models and/or theories. The answers to the research questions can provide input for improving the look and feel and architecture of the technology as well as the fit between the technology, the user, and the context. In turn, this information can be used to increase the effectiveness, persuasiveness, and the long-term usage of the technology.

### Data Collection

Depending on the research questions, there are different ways to collect log data. To gain rich and in-depth knowledge regarding the usage patterns of individual users server-side log data, containing information about communications with the server (requests such as opening a page, clicking a link, saving health values, or other information), can be collected. In Web-based applications, this type of logging is preferably a file where the Web addresses of the requested subpages of the system are registered. This is the most efficient way without substantially losing system performance. Another advantage is that, after updates and modifications of the system, Web addresses referring to new subpages and functions are automatically logged.

On the other side, a single user action (eg, clicking a button to add a health measurement) can lead to multiple server requests leading to multiple Web addresses in the log data file. This can make it harder to identify single user actions. It is therefore necessary to determine which (combinations of) server requests specify certain actions and to link these to a specific identification for that action, such as a code or description.

Besides server-side logging, client-side logging can also provide valuable information regarding the usage of the technology. Client-side information contains actions that do not require server requests, such as scrolling up and down the screen, moving the pointer, and clicking and filling out a text field. The research questions determine the logging method that provides the most valuable information. However, it is also important to take the possibilities and the consequences of the different logging methods into account, such as a loss of system performance.

#### Requirements of Log Data

**Figure 2:**
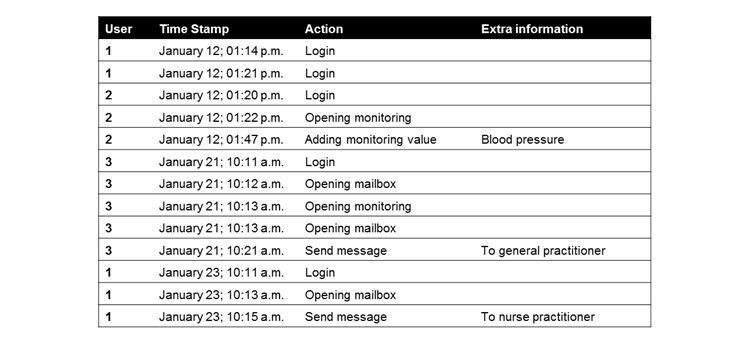
A fictional example of log data.

Log data files are most often Comma-Separated Values (.csv) files that can easily be opened using Excel or SPSS. Information regarding the user identity, date and time of the action, and an identification of the action is essential to identify the user, logins, and the usage patterns within and between logins. An example of a fictional log data file is shown in Figure 2. Depending on the research questions, additional information can be desirable regarding the device (eg, personal computer, mobile phone, tablet, or wearable), a specification of the action (eg, measurements or other information saved in the database), the Global Positioning System (GPS) coordinates of the user, and the status of the user collected via wearables, such as stress, sleep, and activity patterns.

In order to answer the research questions, data files should be of sufficient quality, wherein the goal of the technology and the used behavior change theories and models form the basis for the data that is needed for the analyses. For example, if the research question is “What are predictors for adherence to the technology?” then the data should contain information from which the adherence can be derived, as well as the variables (such as user or usage characteristics) that might possibly predict adherence. When the focus is on exploring the dose-response relationship, there must be a possibility to link the log data to other outcomes, for instance, via the user identification number.

The amount of data needed depends on the complexity of the research question and can only be determined empirically. In general, a reasonable amount of data per user and a reasonable amount of users are needed in the dataset. For example, when analyzing 100 usage sessions, 10 usage sessions of 10 different users provide more generalizeable information than 50 usage sessions of 2 different users. Of course, the more data the better, but it needs to contain the needed information to answer the research question as well.

Importantly, the data should be available for analysis under the applicable privacy regulations, whether or not with informed consent of the individual users of the technology. Informed consent depends upon whether log data includes or needs to be combined with personal data. Currently, it remains undecided whether log data in itself is personal data, as usage data does not always, per se, contain information that can be traced back to individual users. However, as the possibilities for data analytics develop, it may become quite possible in the (near) future to trace users back to specific individuals based on their usage patterns on other technologies. Narayanan and Shmatikov, for example, were able to de-anonymize Netflix users based on reviews in the Internet Movie Database (IMDB) [27]. Thus, it may be hard to truly anonymize log data. Therefore, researchers need to always consider the applicable regulations to determine whether informed consent is needed from the users before log data can be collected and analyzed for research purposes.

#### Data Preparation

**Figure 3:**
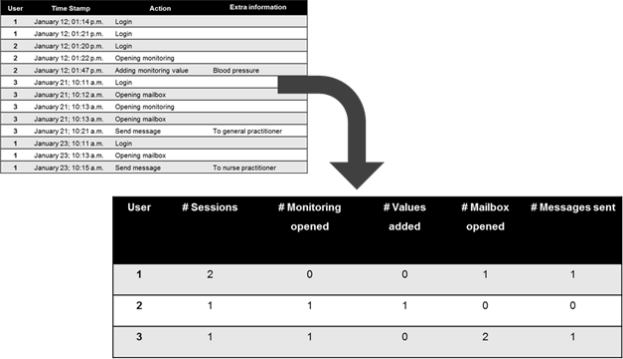
An example of data transformation, based on the data in Figure 2.

Preparing the data before analysis is vital since, for the most part, typical log data consist of tens of thousands records. Hence, these records must first be filtered for the information that is needed for the analysis (eg, Web addresses or the codes for specific actions). Then this information needs to be translated into new variables, such as the number of sessions and/or activities per user, sequence of the activities, and/or time spent per login. An example of how the raw data depicted in Figure 2 can be translated into data for analyses is shown in Figure 3.

In Figure 3, every row in the new data set represents one user. The number of sessions is defined as a period of activity ended by a period of at least 30 minutes of inactivity. In this example, this definition has consequences for User 1, having 3 logins and 2 sessions. By counting the number of logins it would seem like this user has a higher activity level then by counting the number of sessions. It is possible that a user picks up the activities where he left off when returning within 30 minutes after the last action. Furthermore, no user actions were registered during the first and the second login. Hence, counting the number of logins (only) might give a distorted image on the amount of actual use of the different elements of technology.

Second, a distinction was made in this example between visiting a certain element of the technology and actually using it (eg, opening, monitoring, and adding a value, or opening the mailbox and sending a message). Thus, 2 out of the 3 users in this example opened the function for monitoring, but only 1 actually used this function by adding a value to the database. Furthermore, 2 out of 3 users opened their mailboxes and sent a message to a caregiver, where User 3 opened the mailbox twice but sent a message only once.

These are only a few examples of the variables that can be calculated from a raw log data set. Depending on the research questions, new or other variables can be calculated as well. When the question is how the usage in the first sessions correlates to adherence, a distinction can be made between the activities (eg, opening the mailbox and sending a message) in the first, second, third, and further sessions. Also, variables can be added regarding the adherence by a user (where an adherent user is indicated with a “1” and a non-adherent user with a “0”), whether the sent message has been answered (and within what time period), the location of the user (where GPS coordinates are given for every session), the emotional status of the user, activity levels, or the time (in days or hours) between the previous and the current sessions. By combining different data sources, new variables do not have to contain, by definition, information from the same data file.

### Data Analysis

Once the log datasets are prepared, the files are ready for analysis. The first, and up to now, most commonly used method is to describe the frequencies of use, including number of logins or sessions per user, use of the different elements of the technology, moment of use, and time spent on the technology. Although more exposure to an eHealth technology does not always lead to better health outcomes, this information might still provide a starting point for further research. Next, pathway analyses and predictive modeling provide deeper insights into the usage patterns of individual users.

A pathway analysis can provide more information regarding the different usage patterns that occur. In previous research, for example, the usage patterns of adherers and early and late non-adherers to the ”Living to the Full“ intervention were compared [21]. A 1-way analyses of variance (ANOVA) and chi-square tests showed that early non-adherers used fewer and shorter sessions than late non-adherers and adherers. Early non-adherers also used fewer sessions to complete a lesson. Furthermore, late non-adherers had a shorter total duration of sessions than the adherers. Logistic regression was used to assess the baseline characteristics of adherers and non-adherers.

We have also analyzed usage patterns of first visits to the PHR for patients with chronic conditions [23]. The results showed that users followed the structure of the system. While these analyses were conducted by hand on a subset of all users, methods for Markov modeling can be more convenient for analyzing the dominant path through the system of a more extended group of users [28,29]. This methodology can be used, for example, to analyze how people use the different elements of a technology in terms of frequency and the order they select to reach a certain goal [30].

More advanced and predictive information for pattern recognition within complex data sets can be obtained by applying machine-learning algorithms [31]. To do so, the Waikato Environment for Knowledge Analysis (Weka) tool is a relatively accessible and easy to use software package for applying machine learning algorithms for data mining tasks [32]. By using Weka, supervised learning, unsupervised learning, and market-basket methods for analysis can be applied. Supervised learning predicts adherence and effects from early use patterns, which enables early intervention for users at risk [31,33,34]. This method has the potential to answer research questions concerning when users drop out and what the predictors are for users dropping out or returning to the application. Unsupervised learning determines what usage profiles appear from the log data and if this data can be matched to a certain group of participants [33,35]. This method has the potential to answer research questions like ”What are the characteristics of (non-)users, and who are the dropouts?“ Market-basket analysis allows researchers to ask what elements of the technology are often used together [36]. Examples of such analyses can be found in the domain of online shopping, where customers get to see suggestions of products based on the products they looked at.

Although it is difficult to make predictions based on the usage data of relatively small groups of users (eg, in a RCT) and not all research questions can be answered with this data, even these analyses can provide valuable scientific and practical input for future system improvements.

## Discussion

Log data analyses can be used as part of the formative, as well as the summative, evaluation of eHealth technology. As a formative evaluation, log data can provide ongoing and real-time information on how to improve the technology and on the process in which the technology is embedded. As part of the summative evaluation, log data can provide explanations on the uptake and the outcomes of the technology, which can be both scientifically and practically valuable.

### Scientific Translation

Log data analyses provide input for opening the black box of eHealth. Log data analyses not only provide insight into the effects of the single elements of a technology, but can also stipulate essential information about the effects of combinations of elements. In this way, log data can provide input to better understand the results of experimental research designs, such as RCTs or the multiphase optimization strategy (MOST) for eHealth evaluation. In a MOST, potential effective elements are selected for incorporation in an intervention (based on existing theories and/or previous research) and tested in 3 subsequent phases (screening phase, refining phase, confirming phase) [37]. Log data has the potential to validate the results of a RCT or these different phases of the MOST. For example, did the users actually find all the incorporated elements of the technology and are these elements used in the intended way? [24] And how does the use of the different elements correlate to the found effects, and for whom? Based on such insights, existing technologies can be improved and effective elements can be identified and combined into new technologies. These results are not always revealed through questionnaires, interviews, or usability tests.

Another advantage of a log data analysis is that it can reveal real-time insights into the user’s response to specific persuasive triggers in different situations (eg, in terms of location, status of the user), providing new possibilities for the timing of persuasion [38]. Furthermore, several studies have demonstrated that individuals respond differently to the same persuasive strategies [39], indicating that personalization of a technology (adapting a technology to individual users) might increase a program’s persuasiveness and its long-term use and effectiveness [40].

In the domain of eHealth, personalization is often limited to adapting the content of the technology to a confined user profile based on user characteristics like age, gender, and level of education [41]. However, there is evidence that such demographics (user profiles) do not predict engagement [42]. User profiles, such as early usage patterns for example, do potentially predict whether or not an individual will maintain long-term use of an application. For example, Freyne et al [25] found that individuals’ use of specific elements of a technology in the first week influences their use of that technology in the second week. Based upon these results, more extended user profiles can be created that take (early) usage behavior into account, extending user profiles beyond a limited set of user characteristics.

Log data analyses also allow a timely response to flaws in the technology, a shorter evaluation cycle, and the development of more transparent technology, as stressed in the ”agile science“ approach [7]. Until now, modifications of technologies have often been made after an evaluation period. However, technology use is not stagnant, rather it is dynamic and changing over time, and statistics that fit this characteristic are required [43]. With a real-time analysis of log data, adaptive interfaces can be created that respond to individual (changes in) usage patterns. The effects of these adaptive interfaces on the usage can then be analyzed further and improved.

Log data can also be used to test the models and theories that are incorporated in the technology in order to improve the existing behavioral models. Patrick and colleagues made the following comparison for this approach:

It could be argued that today’s current behavioral theories are akin to the Farmer’s Almanac as they are largely descriptive, past-oriented, and simplified to a few elements. These models for understanding behavior and behavior change provide largely “on average” insights without the level of specification and prediction that could occur in behavioral science if the approach to communication, data, and iterative evaluation of computationally complex, multilevel models now common in meteorology could be replicated. (p829) [31]

Log data has the potential to predict usage behavior and can thus be of added value in the development of complex, multilevel models for behavior change.

Furthermore, machine-learning algorithms can make predictions regarding whether and when a user might dropout from using the technology, making it possible to intervene in a timely manner and increase adherence to the technology. However, when focusing on research questions to assess adherence, it is important to substantiate this term: when is a user considered to be adherent? In research, assumptions are made about the intended usage of a technology which are not well defined or evidence-based [44-46]. As a result, it is hard to assess the results of the analyses and compare those to the outcomes of other, similar technologies. A definition of adherence does not always have to be derived from the extent to which a technology is completed (eg, a user is adherent when he/she completed 3 out of 4 lessons), but can also be extracted from other literature. For example, Kaushal and Rhodes discovered that exercising for at least 4 times a week for 6 weeks was the minimum activity to establish an exercise habit [47]. This type of evidence can be used for defining adherence to a technology, for example, a user is adherent when at least 4 usage sessions per week can be identified for a period of 6 weeks. In this example, mere login data (eg, the more logins, the better the adherence is) does not reveal adherence, but assigning a substantiated threshold value does.

An advantage of log data is that it is always available and easy to collect, without requiring any extra effort from the participants. A common problem in (eHealth) research is that participants often find it time consuming and labor intensive to complete questionnaires at different time points or to participate in an interview or focus group, resulting in dropouts from the research study. However, this result does not necessarily mean that the same participants who did not participate in the research dropped out from using the technology. By using log data in addition to questionnaires, researchers have more than one method to collect data and are no longer dependent on having a majority of the participants complete questionnaires or participate in interviews or focus groups.

However, there are important limitations for using log data in eHealth evaluation. First, the results of the log data analyses do not always indicate why certain usage patterns occur. It is therefore important to use a mixed-methods approach to combine the analyses with additional research via interviews, usability tests, or other quantitative and qualitative research methods. For example, the log data analysis from the ”Living to the Full“ intervention showed that a fairly large group dropped out during the 6th lesson. It was revealed by counselors giving the course that this is indeed a hard lesson for participants because of the focus on observing themselves and learning new skills to accept suffering [21]. Additional research can provide more precise insight into what users experience or why they tend to drop out at certain points. Log data analysis focused on such questions can provide researchers specific areas or user groups to examine through future interviews, questionnaires, or usability tests. The results of these evaluations can then be used to improve the technology as well as to highlight the crucial moments in the treatment protocols for blended therapy. Furthermore, using log data in research might require an extra effort from researchers, developers, database managers, etc. For example, it takes time to develop a plan for data collection, management, and analysis, as well as to incorporate the possibility for data collection into the technology.

### Practical Value

Besides the scientific value, the results of a log data analysis can be of added value for eHealth developers and healthcare providers. For example, the results of a pathway analysis and the identified usage profiles can be used as input for adapting and matching the system design to the users in order to make the technology more persuasive. Information regarding the elements that are often used together can also provide real-time feedback and suggestions to the users, guiding their follow-up actions in the system such as:

You have added a goal. Other users have added their current weight as well. Click here to add your weight

Because log data analysis via (un)supervised learning can provide information about users that might potentially drop out from an intervention, on a practical level, healthcare providers can then make use of this information to intervene and stimulate these users to continue using the system. In addition, log data can be used to show healthcare providers how their responsiveness to client messages influences a client’s adherence to the therapy. When composing protocols for (blended) care via eHealth technologies, researchers can then take advantage of the added value of log data analyses.

Until recently, technologies have often changed after an evaluation period, but with a real-time analysis of log data, adaptive interfaces can be created that respond to individual users. The effects of the interface on the use of the technology can then be directly identified, allowing a fast response to flaws in the technology, a shorter evaluation cycle, and the development of more transparent technology.

### Conclusions

The analysis of log data can be of great value for scientists and designers as well as caregivers and policy makers in their research into the black box of eHealth technology. A deliberate analysis of log data can provide insight into the usage of the technology by all users as a group as well as by individual users, helping to accelerate the persuasiveness and effectiveness of eHealth technology. Furthermore, log data can be used to assess the theories that underpin a technology. However, from the collection of log data to translating the results into valuable information, various steps need to be taken, each with their own considerations. This paper serves as a starting point for using log data analysis in eHealth research.

## Abbreviations

ACT: acceptance and commitment therapy
eHealth: electronic health
GPS: Global Positioning System
MOST: multiphase optimization strategy
PHR: personal health record
RCT: randomized controlled trial
Weka: Waikato Environment for Knowledge Analysis

## References

[1] Oinas-Kukkonen H, Harjumaa M (2009). Persuasive systems design: key issues, process model, and system features. CAIS.

[2] Kelders SM, Oinas-Kukkonen H, Oörni Anssi, van Gemert-Pijnen JE (2016). Health behavior change support systems as a research discipline; a viewpoint. Int J Med Inform.

[3] Nijland N, van Gemert-Pijnen JE, Kelders SM, Brandenburg BJ, Seydel ER (2011). Factors influencing the use of a Web-based application for supporting the self-care of patients with type 2 diabetes: a longitudinal study. J Med Internet Res.

[4] van Gemert-Pijnen JE, Nijland N, van Limburg M, Ossebaard HC, Kelders SM, Eysenbach G, Seydel ER (2011). A holistic framework to improve the uptake and impact of eHealth technologies. J Med Internet Res.

[5] Black AD, Car J, Pagliari C, Anandan C, Cresswell K, Bokun T, McKinstry B, Procter R, Majeed A, Sheikh A (2011). The impact of eHealth on the quality and safety of health care: a systematic overview. PLoS Med.

[6] Eysenbach G, CONSORT-EHEALTH Group (2011). CONSORT-EHEALTH: improving and standardizing evaluation reports of Web-based and mobile health interventions. J Med Internet Res.

[7] Hekler EB, Klasnja P, Riley WT, Buman MP, Huberty J, Rivera DE, Martin CA (2016). Agile science: creating useful products for behavior change in the real world. Transl Behav Med.

[8] Moller AC, Merchant G, Conroy DE, West R, Hekler E, Kugler KC, Michie S (2017). Applying and advancing behavior change theories and techniques in the context of a digital health revolution: proposals for more effectively realizing untapped potential. J Behav Med.

[9] Han JY (2011). Transaction logfile analysis in health communication research: challenges and opportunities. Patient Educ Couns.

[10] Resnicow K, Strecher V, Couper M, Chua H, Little R, Nair V, Polk TA, Atienza AA (2010). Methodologic and design issues in patient-centered e-health research. Am J Prev Med.

[11] van Gemert-Pijnen JE, van Gemert-Pijnen JE, Peters O, Ossebaard HC (2013). Improving eHealth.

[12] Bruckman A, Weiss J, Nolan J, Hunsinger J, Trifonas P (2006). Analysis of log file data to understand behavior learning in an online community. International Handbook of Virtual Learning Environments.

[13] Jamali HR, Nicholas D, Huntington P (2005). The use and users of scholarly e‐journals: a review of log analysis studies. AP.

[14] Dumais S, Jeffries R, Russell Dm, Tang D, Teevan J, Olson JS, Kellogg WA (2014). Understanding user behavior through log data analysis. Ways of Knowing in HCI.

[15] Jansen BJ (2006). Search log analysis: What it is, what's been done, how to do it. Libr Inf Sci Res.

[16] Choi Y, Chang S, Kim Y, Lee H, Son W, Jin S (2015). Detecting and monitoring game bots based on large-scale user-behavior log data analysis in multiplayer online games. J Supercomput.

[17] Chatterjee S, Price A (2009). Healthy living with persuasive technologies: framework, issues, and challenges. J Am Med Inform Assoc.

[18] Glasgow RE, Christiansen SM, Kurz D, King DK, Woolley T, Faber AJ, Estabrooks PA, Strycker L, Toobert D, Dickman J (2011). Engagement in a diabetes self-management website: usage patterns and generalizability of program use. J Med Internet Res.

[19] Kim E, Stolyar A, Lober WB, Herbaugh AL, Shinstrom SE, Zierler BK, Soh CB, Kim Y (2007). Usage patterns of a personal health record by elderly and disabled users. AMIA Annu Symp Proc.

[20] Kuijpers W, Groen WG, Oldenburg HS, Wouters MW, Aaronson NK, van Harten WH (2016). eHealth for breast cancer survivors: use, feasibility and impact of an interactive portal. JMIR Cancer.

[21] Kelders SM, Bohlmeijer ET, van Gemert-Pijnen JE (2013). Participants, usage, and use patterns of a web-based intervention for the prevention of depression within a randomized controlled trial. J Med Internet Res.

[22] Pagliari C, Detmer D, Singleton P (2007). Potential of electronic personal health records. BMJ.

[23] Sieverink F, Kelders SM, Braakman-Jansen LMA, van Gemert-Pijnen JE (2014). The added value of log file analyses of the use of a personal health record for patients with type 2 diabetes mellitus: preliminary results. J Diabetes Sci Technol.

[24] Van Gemert-Pijnen JE, Kelders SM, Bohlmeijer ET (2014). Understanding the usage of content in a mental health intervention for depression: an analysis of log data. J Med Internet Res.

[25] Freyne J, Saunders I, Brindal E, Berkovsky S, Smith G (2012). Factors associated with persistent participation in an online diet intervention. CHI '12 Extended Abstracts on Human Factors in Computing Systems.

[26] Bohlmeijer E, Fledderus M, Rokx T, Pieterse M (2011). Efficacy of an early intervention based on acceptance and commitment therapy for adults with depressive symptomatology: evaluation in a randomized controlled trial. Behav Res Ther.

[27] Narayanan A, Shmatikov V (2008). Robust de-anonymization of large sparse datasets.

[28] Seneta E (1996). Markov and the birth of chain dependence theory. Int Stat Rev.

[29] Borges J, Levene M (2007). Evaluating variable-length Markov chain models for analysis of user web navigation sessions. IEEE Trans Knowl Data Eng.

[30] Akkersdijk SM, Kelders SM, Braakman-Jansen LMA, van Gemert-Pijnen JE (2017). Using Markov chains to analyze paths through a personal health record. Persuasive Technology XII (Adjunct Proceedings).

[31] Patrick K, Hekler EB, Estrin D, Mohr DC, Riper H, Crane D, Godino J, Riley WT (2016). The pace of technologic change: implications for digital health behavior intervention research. Am J Prev Med.

[32] Hall M, Frank E, Holmes G, Pfahringer B, Reutemann P, Witten IH (2009). The WEKA data mining software. SIGKDD Explor Newsl.

[33] Han J, Pei J, Kamber M (2011). Data Mining: Concepts and Techniques.

[34] Hekler EB, Michie S, Pavel M, Rivera DE, Collins LM, Jimison HB, Garnett C, Parral S, Spruijt-Metz D (2016). Advancing models and theories for digital behavior change interventions. Am J Prev Med.

[35] Paliouras G, Papatheodorou C, Karkaletsis V, Spyropoulos C (2002). Discovering user communities on the Internet using unsupervised machine learning techniques. Interact Comput.

[36] Anand S, Patrick A, Hughes J, Bell D (1998). A data mining methodology for cross-sales. Knowl Based Syst.

[37] Collins LM, Murphy SA, Strecher V (2007). The multiphase optimization strategy (MOST) and the sequential multiple assignment randomized trial (SMART): new methods for more potent eHealth interventions. Am J Prev Med.

[38] Räisänen T, Oinas-Kukkonen H, Pahnila S (2008). Finding kairos in quitting smoking: smokers' perceptions of warning pictures. Persuasive Technology: Third International Conference.

[39] Kaptein M, Eckles D (2012). Heterogeneity in the effects of online persuasion. JIM.

[40] Kaptein M, Markopoulos P, de Ruyter B, Aarts E (2015). Personalizing persuasive technologies: explicit and implicit personalization using persuasion profiles. Int J Hum Comput Stud.

[41] Kaptein M, Lacroix J, Saini P (2010). Individual Differences in Persuadability in the Health Promotion Domain. PERSUASIVE 2010.

[42] van Mierlo T, Li X, Hyatt D, Ching AT (2017). Demographic and indication-specific characteristics have limited association with social network engagement: evidence from 24,954 members of four health care support groups. J Med Internet Res.

[43] Magni M, Susan Taylor M, Venkatesh V (2010). ‘To play or not to play’: a cross-temporal investigation using hedonic and instrumental perspectives to explain user intentions to explore a technology. Int J Hum Comput Stud.

[44] Kelders SM, Kok RN, Ossebaard HC, Van Gemert-Pijnen JE (2012). Persuasive system design does matter: a systematic review of adherence to web-based interventions. J Med Internet Res.

[45] Donkin L, Christensen H, Naismith SL, Neal B, Hickie IB, Glozier N (2011). A systematic review of the impact of adherence on the effectiveness of e-therapies. J Med Internet Res.

[46] Su W, Chih M (2016). Is more eHealth system use better for cancer patients and family caregivers? A literature review.

[47] Kaushal N, Rhodes RE (2015). Exercise habit formation in new gym members: a longitudinal study. J Behav Med.

