# Correction for: Opening the Black Box of Electronic Health: Collecting, Analyzing, and Interpreting Log Data

**DOI:** 10.2196/resprot.8879

**Published:** 2018-01-17

**Authors:** Floor Sieverink, Saskia Kelders, Mannes Poel, Lisette van Gemert-Pijnen

**Affiliations:** ^1^ Centre for eHealth and Wellbeing Research Department of Psychology, Health and Technology University of Twente Enschede Netherlands; ^2^ Optentia Research Focus Area North-West University Vanderbijlpark South Africa; ^3^ Human Media Interaction Group Department of Computer Science University of Twente Enschede Netherlands

The authors of “Opening the Black Box of Electronic Health: Collecting, Analyzing, and Interpreting Log Data” (JMIR Res Protoc 2017;6(8):e156) would like to make a correction to Reference 42. The first author’s name was incorrectly rendered as “van MT”. The full reference should read:

42. van Mierlo T, Li X, Hyatt D, Ching AT. Demographic and indication-specific characteristics have limited association with social network engagement: evidence from 24,954 members of four health care support groups. J Med Internet Res 2017 Feb 17;19(2):e40

The corrected article will appear in the online version of the paper on the JMIR website on January 17, 2017, together with the publication of this correction notice. Because this was made after submission to PubMed or Pubmed Central and other full-text repositories, the corrected article also has been re-submitted to those repositories.

